# Medical Staff and Resident Preferences for Using Deep Learning in Eye Disease Screening: Discrete Choice Experiment

**DOI:** 10.2196/40249

**Published:** 2022-09-20

**Authors:** Senlin Lin, Liping Li, Haidong Zou, Yi Xu, Lina Lu

**Affiliations:** 1 Shanghai Eye Disease Prevention and Treatment Center, Shanghai Eye Hospital Shanghai China; 2 Shanghai Key Laboratory of Ocular Fundus Diseases, Shanghai General Hospital, Shanghai Engineering Center for Visual Science and Photomedicine Shanghai China; 3 Shanghai Hongkou Center for Disease Control and Prevention Shanghai China

**Keywords:** discrete choice experiment, preference, artificial intelligence, AI, vision health, screening

## Abstract

**Background:**

Deep learning–assisted eye disease diagnosis technology is increasingly applied in eye disease screening. However, no research has suggested the prerequisites for health care service providers and residents willing to use it.

**Objective:**

The aim of this paper is to reveal the preferences of health care service providers and residents for using artificial intelligence (AI) in community-based eye disease screening, particularly their preference for accuracy.

**Methods:**

Discrete choice experiments for health care providers and residents were conducted in Shanghai, China. In total, 34 medical institutions with adequate AI-assisted screening experience participated. A total of 39 medical staff and 318 residents were asked to answer the questionnaire and make a trade-off among alternative screening strategies with different attributes, including missed diagnosis rate, overdiagnosis rate, screening result feedback efficiency, level of ophthalmologist involvement, organizational form, cost, and screening result feedback form. Conditional logit models with the stepwise selection method were used to estimate the preferences.

**Results:**

Medical staff preferred high accuracy: The specificity of deep learning models should be more than 90% (odds ratio [OR]=0.61 for 10% overdiagnosis; *P*<.001), which was much higher than the Food and Drug Administration standards. However, accuracy was not the residents’ preference. Rather, they preferred to have the doctors involved in the screening process. In addition, when compared with a fully manual diagnosis, AI technology was more favored by the medical staff (OR=2.08 for semiautomated AI model and OR=2.39 for fully automated AI model; *P*<.001), while the residents were in disfavor of the AI technology without doctors’ supervision (OR=0.24; *P*<.001).

**Conclusions:**

Deep learning model under doctors’ supervision is strongly recommended, and the specificity of the model should be more than 90%. In addition, digital transformation should help medical staff move away from heavy and repetitive work and spend more time on communicating with residents.

## Introduction

Vision loss, defined as either visual impairment or blindness, is becoming a vital aspect of public health [1], affecting economic, educational, and employment opportunities, reducing the quality of life, and increasing the risk of death [1]. Therefore, according to the recent eye care competency framework by the World Health Organization, the continuum of eye care across all levels of the health system should be highlighted, particularly primary health care, to support universal health coverage [2].

High-quality eye disease prevention health care, such as effective screening, can help eliminate almost 57% of all blindness cases [3]. Nowadays, artificial intelligence (AI) is gradually adopted in eye disease screening and may assist in addressing the limited and difficult-to-sustain resources in screening capacity, personnel costs, and diagnosis expertise [4]. The accuracy of AI models greatly affects the cost-effectiveness of eye disease screening [5]. Unfortunately, though the US Food and Drug Administration (FDA) had set a mandatory level of accuracy with a sensitivity of more than 85% and a specificity of more than 82.5% [6], the accuracy of AI-assisted eye disease screening systems in the real world were far worse than that reported in the model development phase [7]. Therefore, it is essential to make clear the medical staff and resident requirements of the accuracy of AI models in the community-based eye disease screening in the real world. However, no related research has been conducted thus far.

To fill this evidence gap, we conducted discrete choice experiments (DCEs) for health care providers and residents in Shanghai, China, from August 2021 to January 2022. We aimed to reveal the preferences of medical staff and residents for using AI technology in community-based eye disease screening, particularly their preference for accuracy. The DCE technique, originating in mathematical psychology, has been introduced in health economics to elicit preferences for health and health care [8]. Additionally, the DCE technique is predictive of choices, mimicking real-world decisions in health care decision-making (correctly predicting >93% of choices) [9].

## Methods

### Study Setting

Shanghai, with a population of 24 million in 2019, is the economic, science, and technology innovation center in China. It is also one of the first cities in the world to adopt deep learning (DL) models to establish affordable and sustainable community-based eye disease screening systems. Since 2015, a teleophthalmology-based eye disease screening system covering all community health service centers has been developed in Shanghai. Residents can take free fundus photographs once a year by the trained general practitioners (GPs) in community health service centers. The fundus photos are then sent to the designated eye disease diagnosis centers through a dedicated information system. After the ophthalmologists in the diagnosis centers read the fundus photos and make diagnoses, the screening results are returned to the community health service centers. The GPs may inform residents of the screening results and provide medical advice.

**Figure 1:**
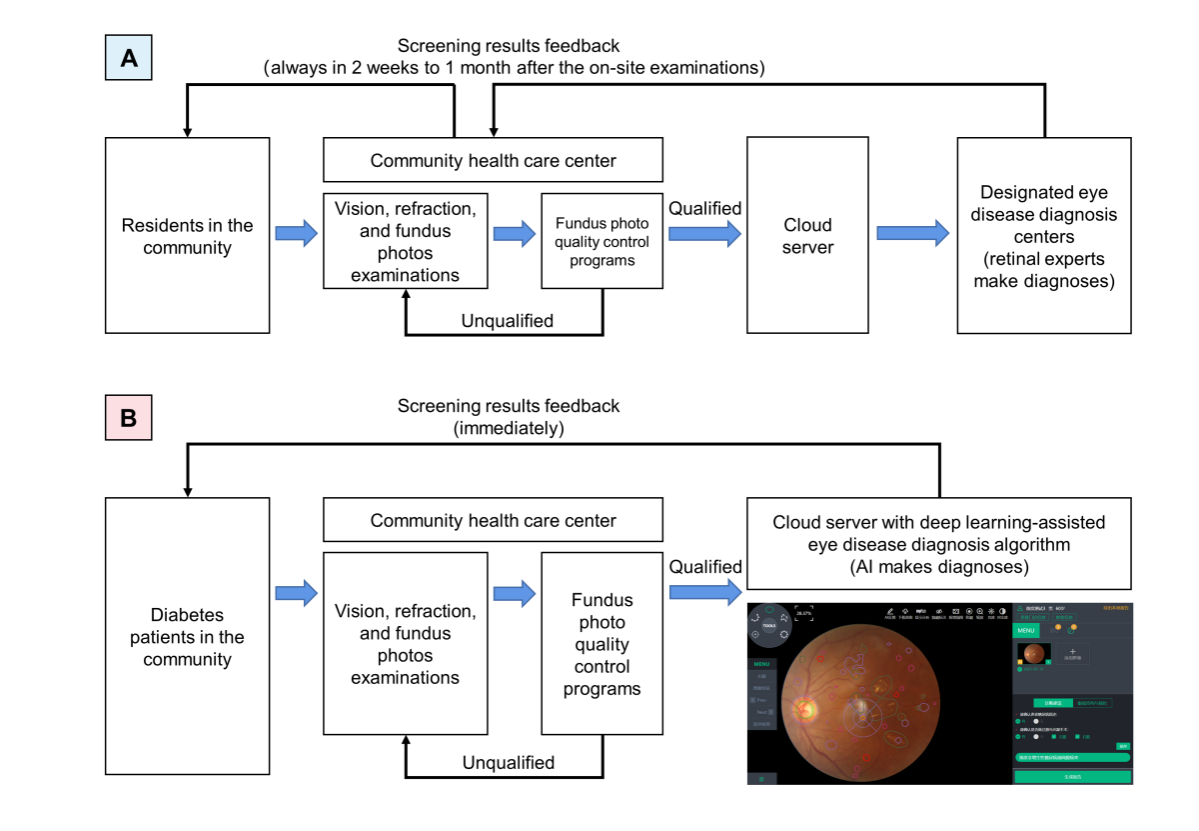
Process of community-based eye disease screening in Shanghai. A: teleophthalmology-based eye disease screening system; B: deep learning–assisted eye disease screening system. The photograph in the lower right corner of process B is a sample of the operation interface of the deep learning–assisted eye disease diagnosis system. AI: artificial intelligence.

In 2020, an AI-assisted eye disease screening system was established using DL model on cloud servers instead of ophthalmologists in the diagnosis centers making the screening diagnoses (Figure 1) [10-12]. The accuracy of DL models used for community-based eye disease screening has been reported widely [6,11,13,14]. Thus far, 56 community health service centers have shifted to the AI-assisted eye disease screening system. In 2021, these community health service centers screened over 40,000 residents with the help of the DL model and found over 7000 residents with suspected eye diseases.

### Discrete Choice Experiments and Participants Inclusion

We conducted 2 DCEs to assess medical staff’s preferences (experiment 1) and residents’ preferences (experiment 2) for using the DL model in community-based eye disease screening. The main reason for using a DCE is that simply asking the respondents to rate the screening strategy attributes or choose their preferred item from a scale generally yields no more information than the fact that they want all the benefits and none of the indirect or direct costs [15]. Choosing between alternatives forces them to make a trade-off and choose, as in real life, between options that may increase utility (eg, improved diagnosis accuracy) and decrease utility (eg, screening cost of 40 CNY [US $6.15] per resident instead of being free).

**Figure 2:**
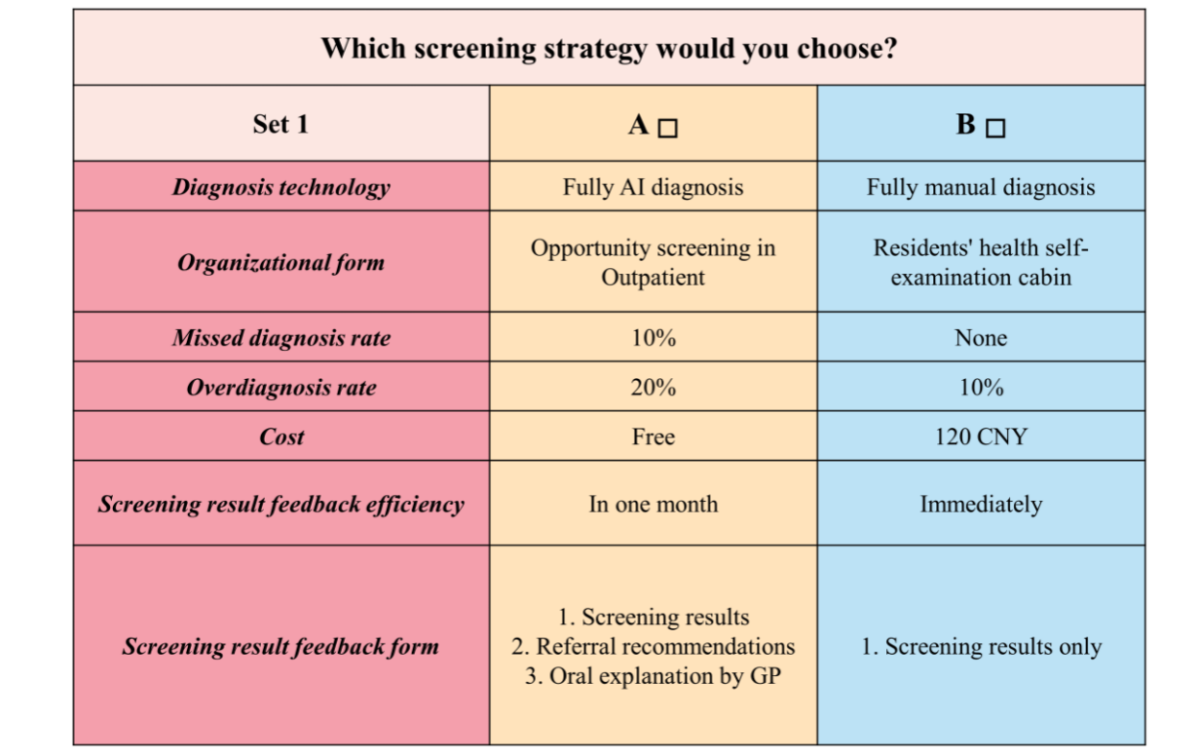
Example of the choice sets applied. Both options include the same 7 attributes. Health care service providers and residents were asked to decide between options A and B (in 2021, 1 USD=6.5 CNY). AI: artificial intelligence; GP: general practitioner.

Based on previously published literature [4-7,16-18], 4 attributes were identified initially to describe the outline of the community-based eye disease screening, including the accuracy, screening result feedback efficiency, level of ophthalmologist’s involvement, and cost. It was worth stating that “screening result feedback efficiency” was included in the attributes because nearly instantaneous feedback might increase compliance [18]; moreover, “level of ophthalmologists involvement” was included because algorithmic aversion might exist [19]. To assess the appropriateness of these potential attributes and their levels, 5 experts on eye care were interviewed face-to-face in the Shanghai Eye Disease Control and Treatment Center. Based on these interviews, the attribute *accuracy* was divided into the following 2 attributes: “missed diagnosis rate” and “overdiagnosis rate,” as they might have different impacts on the acceptability of eye disease screening. In addition, 2 new attributes were added: “organizational form” and “screening result feedback form,” as the adoption of the DL model had the potential to reform the screening programs. As a result, 7 attributes were used to describe the outline of the community-based eye disease screening, and each attribute was divided into 3-6 levels (Table 1). Three SAS (SAS Institute Inc) procedures—“%mktruns,” “%mktex,” and “%choiceff”—were used to develop the questionnaire [20]. The questionnaire consisted of the following two parts: the respondent’s basic information, such as sex and age, and a few choice sets, each of which contained 2 options with different screening attribute levels (Figure 2). The respondents were asked to choose the more favorable option in each choice set, and they were not allowed to choose both or neither in a set [21].

**Figure 3:**
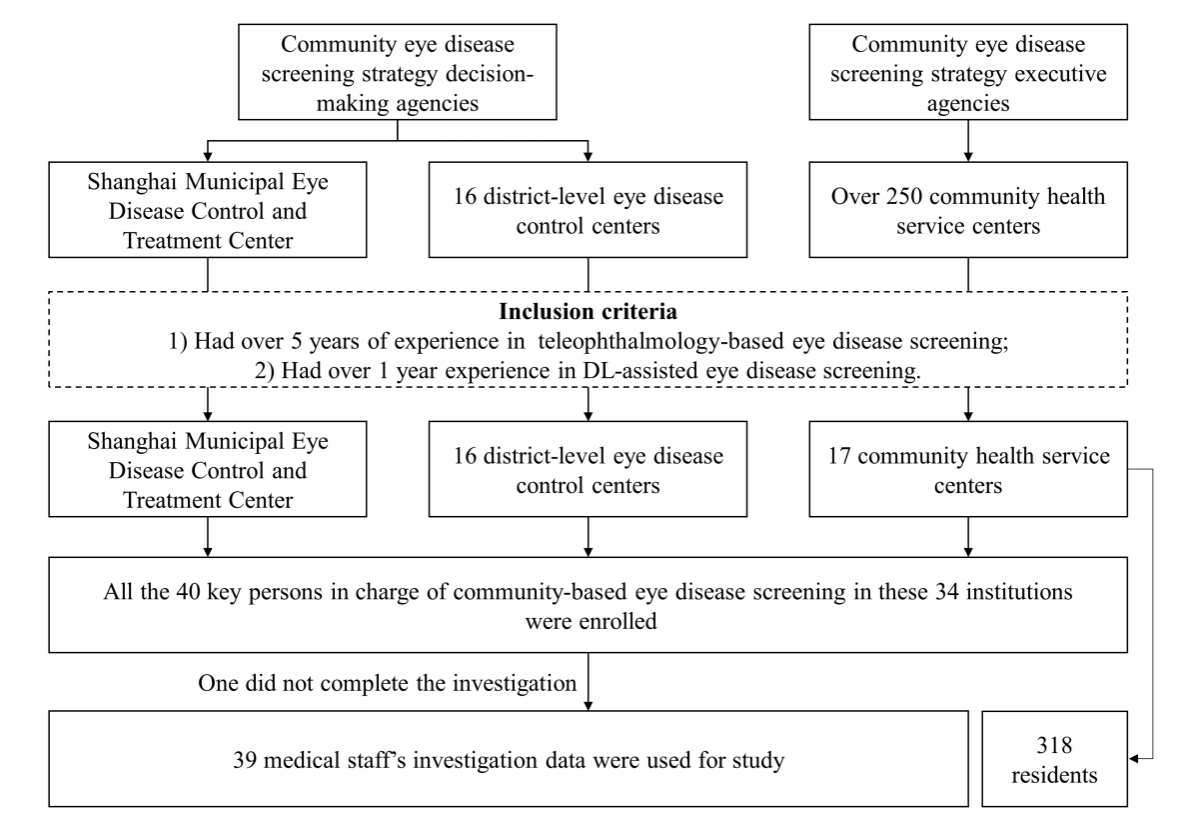
Medical staff and residents inclusion process. DL: deep learning.

In Experiment 1, one municipal and 16 district-level eye disease control centers and over 250 community health service centers in Shanghai were enrolled. To receive rational rather than imaginary choices, the following two strict inclusion criteria were set: (1) they had over 5 years of experience in teleophthalmology-based eye disease screening and (2) they had over 1 year of experience in DL-assisted eye disease screening. A total of 34 institutions met the criteria, including 1 (3%) municipal, 16 (47%) district-level eye disease control centers, and 17 (50%) community health service centers (Figure 3). All the 40 key persons in charge of community-based eye disease screening in these 34 institutions were invited and agreed to participate in the experiment. Due to the limited number of respondents, we had to ask each one to answer a relatively large number of questions. According to the rule of thumb, as proposed by Johnson and Orme [22], we divided the alternative screening strategies into 30 choice sets of 2 options to ensure that the sample size of 40 people met the statistical requirements. The experiment was conducted in the form of a self-administered questionnaire, with a trained investigator on standby to interpret the questionnaire. One respondent quit because of temporary work arrangements. Therefore, data from 39 medical staff were available in the final analysis.

In Experiment 2, we randomly selected 2 from the 17 community health service centers involved in Experiment 1 and conducted the residents’ investigation when carrying out the AI-assisted community-based eye disease screening. All the residents who participated in the screening were invited to the experiment. Because the number of residents was relatively large, we divided the alternative screening strategies into 10 choice sets of 2 options to reduce the response burden for each respondent. According to the rule of thumb, as proposed by Johnson and Orme [22], the minimum of the required sample size was 125. A total of 318 residents were investigated (Figure 3). To help the residents understand the questionnaire, the experiment was conducted using face-to-face questioning by trained investigator.

**Table 1:** Attributes and levels in the discrete choice experiments.

Attributes		Levels					
		1	2	3	4	5	6
**Performance expectancy**							
	Missed diagnosis rate (%)	None	5	10	15	20	—^a^
	Overdiagnosis rate (%)	None	5	10	15	20	—
	Screening result feedback efficiency	Immediately	In 2 weeks	In 1 month	—	—	—
**Effort expectancy**							
	Level of ophthalmologist involvement	Fully automated^b^ DL^c^ model	Semiautomated^d^ DL model	Fully manual diagnosis^e^	—	—	—
**Facilitating conditions**							
	Organizational form	Centralized screening^f^	Residents’ health self-examination cabin^g^	Opportunity screening in outpatient^h^	—	—	—
	Cost	Free	40 CNY^i^	80 CNY	120 CNY	160 CNY	200 CNY
	Screening result feedback form	Screening results^j^	Screening results and medical advice^k^	Screening results, medical advice, and oral explanation by GP^l,m^	—	—	—

^a^Not available.

^b^The screening results were provided entirely by the deep learning model, and the ophthalmologists were not involved in the diagnostic process.

^c^DL: deep learning.

^d^The deep learning model performed the initial screening of fundus photographs and then the ophthalmologists reviewed the results.

^e^The screening results were provided entirely by the ophthalmologists and the deep learning model was not involved in the diagnostic process.

^f^The community health service center informed the residents to undergo the screening at a uniform place and time.

^g^The equipment needed for screening was placed in a specific cabin in the community health service center, and residents could go to the cabin for self-examination at any time.

^h^Residents with chronic diseases and other risk factors would be recommended by general practitioners for eye disease screening during their outpatient follow-up.

^i^US 1$=6.5 CNY.

^j^The report with only the screening results would be given to the residents without any recommendations or explanations.

^k^The report with the screening results and referral recommendations would be given to the residents without explanations.

^l^Besides the report with the screening results and referral recommendations that would be given to the residents, a general practitioner would also explain the meaning of the report.

^m^GP: general practitioner.

### Statistical Analyses

Mean, median, and standard deviation were calculated for the quantitative variables. For categorical variables, the number in a specific category was calculated as a percentage. Pearson chi-square test for nominal variables and Mann-Whitney *U* test for continuous variables were used for statistical analysis. Conditional logit models with the stepwise selection method were used to explore the significant preferences for each attribute level, with the choice responses as the binary dependent variable and the difference in levels for each of the attributes as the independent variables [21]. Two models were used to estimate the medical staff’s and residents’ preference respectively, expressed as odds ratios (ORs) for each attribute level. SAS 9.4 (SAS Institute Inc) were used for statistical analysis. The level of significance was set at *P*<.05.

### Ethics Approval

All participants were adults. Written informed consent from all participants was obtained before enrollment. The study adhered to the principles of the Declaration of Helsinki on Ethics. This study was approved by the Shanghai General Hospital Ethics Committee (2022SQ272).

## Results

The medical staff’s mean age was 39.67 (SD 6.98) years, and they had been responsible for eye disease screening for 6.73 (SD 5.76) years on average. The residents’ mean age was 68.62 (SD 6.96) years; Of the 318 participants, 120 (37.74%) were male and 198 (62.26%) were female. Detailed characteristics of the respondents are shown Table 2.

Table 3 presents the results of the conditional logit models, evaluating the influence of the tested attribute levels on medical staff’s and residents’ preferences. Among the 39 medical staff, the impact of selected attributes on preferences was statistically significant for 4 of the 7 attributes. Generally, medical staff prefer attribute levels with AI technology, lower overdiagnosis rates, lower screening costs, and higher screening result feedback efficiency. The results for the attribute “organizational form,” “missed diagnosis rate,” and “screening result feedback form” were inconclusive—none of the attribute levels were associated with statistically significant utility differences.

Further, we focused on the accuracy of the diagnosis. For the missed diagnosis rate, there were no significant differences of medical staff’s preferences for a missed diagnosis rate between 0% and 20%. However, for the overdiagnosis rate, compared with no overdiagnosis, medical staff’s preference for the 10% overdiagnosis rate significantly decreased (OR=0.61; *P*<.001).

Among the 318 residents, the influence of selected attributes on preferences was statistically significant for 3 of the 7 attributes. Generally, residents were in disfavor of the attribute level with a fully automated DL model (OR=0.24; *P*<.001), but they preferred attribute levels with lower screening costs and oral explanations by GP. The results for the attributes “organizational form,” “missed diagnosis rate,” “overdiagnosis rate,” and “screening result feedback efficiency” were inconclusive. None of the attribute levels were associated with statistically significant utility differences.

**Table 2:** Characteristics of respondents.

Respondent and characteristics			Value
**Medical staff (n=39)**			
	Age (years), mean (SD)		39.67 (6.98)
	**Institution level, n (%)**		
		Municipal eye disease control center	1 (2.56)
		District-level eye disease control center	15 (38.46)^a^
		Community health service center	23 (58.97)
	**Position, n (%)**		
		Institution leader	7 (17.95)
		Department leader	22 (56.41)
		Eye disease screening mainstay	10 (25.64)
	Years in the current position, mean (SD)		6.73 (5.76)
**Resident (n=318)**			
	Age (years), mean (SD)		68.62 (6.96)
	**Sex, n (%)**		
		Male	120 (37.74)
		Female	198 (62.26)
	**Education level, n (%)**		
		Junior high school and below	216 (67.92)
		Senior high school	72 (22.64)
		Junior college	21 (6.6)
		Undergraduate and above	9 (2.83)
	**Eye disease, n (%)**		
		Suspected	73 (22.96)
		None	245 (77.04)

^a^One respondent from a district-level eye disease control center quit the experiment because of temporary work arrangements. Therefore, although 16 district-level eye disease control centers were included in our study, only 15 key persons from these institutions finished the questionnaire.

**Table 3:** Preferences for using deep learning in community-based eye disease screening.

Attribute and level			Medical staff^a^		Residents
			OR^b^ (95% CI)		OR (95% CI)
**Diagnostic technology**					
	Semiautomated DL^c^ model	2.08 (1.71, 2.52)^d^		0.89 (0.68, 1.15)	
	Fully automated DL model	2.39 (1.97, 2.90)^d^		0.24 (0.20, 0.29)^d^	
	Fully manual diagnosis	Reference		Reference	
**Organizational form**					
	Centralized screening	Reference		Reference	
	Residents’ health self-examination cabin	Not significant		Not significant	
	Opportunity screening in outpatient^e^	Not significant		Not significant	
**Missed diagnosis rate**					
	None	Reference		Reference	
	5%	Not significant		Not significant	
	10%	Not significant		Not significant	
	15%	Not significant		Not significant	
	20%	Not significant		Not significant	
**Overdiagnosis rate**					
	None	Reference		Reference	
	5%	0.88 (0.68, 1.15)		Not significant	
	10%	0.61 (0.46, 0.81)^d^		Not significant	
	15%	0.63 (0.48, 0.83)^f^		Not significant	
	20%	0.51 (0.38, 0.68)^d^		Not significant	
**Cost^g^**					
	Free	Reference		Reference	
	40 CNY	0.61 (0.46, 0.83)^f^		0.75 (0.56, 1.01)	
	80 CNY	0.47 (0.35, 0.64)^d^		0.56 (0.42, 0.74)^d^	
	120 CNY	0.39 (0.28, 0.54)^d^		0.82 (0.51, 1.31)	
	160 CNY	0.27 (0.19, 0.38)^d^		0.78 (0.46, 1.32)	
	200 CNY	0.21 (0.15, 0.29)^d^		0.57 (0.46, 0.71)^d^	
**Screening result feedback form**					
	Screening results	Not significant		0.52 (0.44, 0.61)^d^	
	Screening results and referral recommendations	Not significant		0.75 (0.65, 0.87)^d^	
	Screening results, referral recommendations, and oral explanation by GP^h^	Reference		Reference	
**Screening result feedback efficiency**					
	Immediately	Reference		Reference	
	In 2 weeks	0.68 (0.56, 0.82)^d^		Not significant	
	In 1 month	0.58 (0.48, 0.70)^d^		Not significant	

^a^In each grid, an OR value over 1 means that the health care services providers were more inclined to this level, while the value less than 1 means that they disliked this level even more.

^b^OR: odds ratio.

^c^DL: deep learning.

^d^*P*<.001.

^e^Residents with chronic diseases and other risk factors would be recommended by general practitioners for eye disease screening during their outpatient follow-up.

^f^*P*=.001.

^g^In 2021, US $1= 6.5 CNY.

^h^GP: general practitioner.

## Discussion

### Principal Findings

To the best of our knowledge, this study is the first to quantitatively estimate both medical staff’s and residents’ preferences for using DL in community-based eye disease screening in the real world. Since one of the most important questions for achieving universal health coverage in a digital world is whether digital technologies help increase the acceptability of health care services [23,24], our study is significant for the transformation, application, and promotion of this new technology. It was based on the multicenter practices of AI-assisted eye disease screening from 34 medical institutions, where both medical staff and residents under investigation had real service experience of AI. We showed that when compared with a fully manual diagnosis, AI technology was more favored by the medical staff, even after adjusting for the impacts of diagnosis accuracy, cost, and efficiency. However, the residents were in disfavor of the AI technology without doctors’ supervision. Furthermore, to meet the medical staff’s preference, the accuracy of the AI-assisted eye disease screening technology should be much higher than the FDA’s standards. On the contrary, accuracy was not a priority for the residents. They prefer to have the doctors involved in the screening process and leave the choice of accuracy to their general practitioners.

The adoption of DL model for community-based eye disease screening is necessary. Before the development of DL model, the screening relied on ophthalmologists heavily, regardless of conducting traditional face-to-face screening or a telemedicine system [25]. At this stage, continuous eye disease screening was not affordable in most of the countries [6] for two reasons. On the one hand, the limited human resources of the ophthalmologists resulted in extremely high screening costs [5]. On the other hand, the organization of the screening was challenging, requiring the coordination of ophthalmologists, community health centers, and residents at the same time [25]. As a result, in Shanghai, before the adoption of the DL model, each community only could provide screening service to approximately 300 residents per year. On the contrary, after the adoption of DL model, as the ophthalmologist resources were no longer the bottlenecks, the screening use volume dramatically increased to 800 residents per community per year.

Accuracy is regarded as one of the most important considerations in the adoption of DL model. When screening populations with a substantial disease, achieving both high sensitivity and specificity is critical in minimizing both false-positive and false-negative results [26]. The previous studies have shown that it is feasible to meet the mandatory level of accuracy as the primary endpoint with a sensitivity of more than 85% and a specificity of more than 82.5%, which was recommended by the FDA [6,22,27,28]. However, when the DL models were applied in the real world, their accuracy greatly reduced [7]. Therefore, the question is, “what are the medical staff and residents’ requirements of the accuracy of AI models in the real world?”

Our study attempted to answer this question from the perspective of medical staff’s and residents’ preferences in the real-world, community-based eye diseases screening. Although the ideal state is 100% accuracy, under the existing technical conditions, health care service providers must make a trade-off between higher sensitivity and specificity. Both outcomes are important—positive cases should be identified, but this should not come at the cost of overly sensitive screening systems [29].

We showed that if the overdiagnosis rate exceeded 10%, the preferences of the medical staff decreased significantly. Therefore, the specificity of the DL model should be controlled with over 90% accuracy. This does not mean that sensitivity is not important, but rather that the sensitivity standard of the FDA is sufficient. On the one hand, sensitivity is a patient safety criterion, because the primary goal of eye disease screening is to identify the people who are likely to have eye disease and require further evaluation by ophthalmologists [22]. One GP in our study claimed that “the missed diagnosis may harm residents’ trust in eye disease screening and reduce their enthusiasm for screening,” whereas trust acts as a critical element in medical care [30]. On the other hand, the overdiagnosis rate affects the number of residents who receive an unnecessary referral [22]. A higher overdiagnosis rate means more unnecessary specialist visits, which may lead to unnecessary psychological stress for suspected patients and add further referral costs [31]. Therefore, our results indicated that overdiagnosis would cause resentment from both decision-making and executive agencies.

However, though accuracy is critical for medical staff, results show that the residents do not regard it as a priority. They rather focus on whether the doctors are at the center of medical decision-making [32]. Humans are notoriously poor at comprehending probability and evaluating risk, especially when it pertains to their health or the health of a loved one [33]. In the AI era, although medical knowledge—which forms the basis of decision-making—will be as accessible to the patient as the doctor, most patients need a doctor to understand risk and to communicate this to them [33]. Patients look to the doctors for advice when facing uncertainty in their medical decisions [34]. A study of patient attitudes toward AI use has shown that patients felt their doctors should have the final say in their treatment plans to avoid experiencing the potential harm that might result from mistakes made by health care AI [32]. Therefore, AI tools should be used as decision support tools for human diagnosticians, but not in place of them [35].

When it comes to the other attributes, AI technology with a lower cost and higher feedback efficiency is logically preferable. Cost is an important issue in the adoption of AI-assisted eye disease diagnosis technology. Therefore, it is necessary to conduct health economics evaluation [36]. Fortunately, evidence has shown the cost of screening could be saved by using AI technology, which is mainly attributable to the substantial reduction in human assessment time and workforce without sacrificing screening performance [5].

Traditional ophthalmological diagnosis is heavily dependent on the interpretation of images, which is often subjective and qualitative [37]. Reading these images by trained personnel is neither sustainable nor an efficient use of expertise, and AI technology is essential in facilitating the capture, storage, and interpretation of photographs [17]. From the health system’s perspective, the addition of the DL model to fundus photography provides an opportunity to improve this platform for detecting and monitoring retinal diseases on a large scale, and satisfactory results have been obtained [13]. In addition, AI algorithms may bridge the clinical gap [4]. The DL method used for discriminative tasks in ophthalmology, such as diagnosing diabetic retinopathy or age-related macular degeneration, could enhance existing data sets of common and rare ophthalmic diseases without concern for personally identifying information [38]. Other than helping address the limited screening capacity, the DL model may reduce workforce costs and relieve the burden placed on teleophthalmology health care staff [4,39]. The inadequacy of health resources and the vast medical burden may be important reasons for the rapid acceptance the DL method by medical staff.

Regarding feedback efficiency, recent studies have shown that nearly instantaneous feedback may lead to increased patient compliance [5,18]. The most obvious context for the application of AI-assisted diagnosis technology is in primary eye care where the data to be analyzed are complex, the outcomes are simple and well-defined, and the number of people to process is large [18]. In this context, manual diagnosis requires extensive time and energy, whereas AI can work tirelessly and quickly.

### Limitations

The most obvious limitation of our study was that DCE was conducted only in Shanghai. However, as mentioned, Shanghai is one of the pioneers in eye care digital transformation. Therefore, our study is valuable for other regions of the world. The second limitation was that the residents in our experiment were mainly older adults. However, this was consistent with the population that participated in the community-based eye screening in Shanghai because young people mostly participated in physical examinations at their workplace.

### Conclusion

In conclusion, to meet the actual preferences of medical staff and residents for using AI in the community-based eye disease screening, the DL model under doctors’ supervision is strongly recommended, and the specificity of the model should be more than 90%, which is higher than the FDA standard. In addition, digital transformation should help medical staff move away from heavy and repetitive work; however, it should not reduce their involvement in the health care service. Instead, medical staff should spend more time on communicating with residents.

## Abbreviations

AI: artificial intelligence
DCE: discrete choice experiment
DL: deep learning
FDA: Food and Drug Administration
GP: general practitioner
OR: odds ratio

## Availability of Data and Materials

The data sets used and analyzed during this study are available from the corresponding author on reasonable request.
